# Optimization of a MT1-MMP-targeting Peptide and Its Application in Near-infrared Fluorescence Tumor Imaging

**DOI:** 10.1038/s41598-018-28493-9

**Published:** 2018-07-09

**Authors:** Li Ren, Ye Wang, Lei Zhu, Liqiao Shen, Jinrui Zhang, Jingjing Wang, Haolong Li, Qingchuan Zheng, Dahai Yu, Xuexun Fang

**Affiliations:** 1 College of Food Science and Engineering, Jilin University, 5333 Xi’an Street, Changchun, Jilin 130062 P. R. China; 2 State Key Laboratory of Inorganic Synthesis and Preparative Chemistry, Jilin University, 2699 Qianjin Street, Changchun, 130012 P. R. China; 3 School of Life Science, Jilin University, 2699 Qianjin Street, Changchun, Jilin 130012 PR China; 4 Department of Surgery, Emory University School of Medicine, 201 Dowman Drive, Atlanta, GA 30322 United States; 5 Key Laboratory of Molecular Enzymology and Enzyme Engineering of the Ministry of Education, Jilin University, 2699 Qianjin Street, Changchun, Jilin 130012 P. R. China; 6 State Key Laboratory of Molecular Vaccinology and Molecular Diagnostics & Center for Molecular Imaging and Translational Medicine, Xiamen University, Siming South Road, Xiamen, Fujian 361005 P. R. China; 7 Laboratory of Theoretical and Computational Chemistry, Jilin University, Jiefang Road, Changchun Jilin, 130023 P. R. China

## Abstract

Membrane type 1 metalloproteinase (MT1-MMP) is an important regulator of cancer invasion, growth and angiogenesis, thus making it an attractive target for cancer imaging and therapy. A non-substrate peptide (MT1-AF7p) that bonded to the “MT-Loop” region of MT1-MMP was identified by using a phage-displayed peptide library and was used to image the MT1-MMP expression *in vivo* through optical imaging. However, the substrate in the screening did not have a 3D structure, thus resulting in a loose bonding of MT1-AF7p. To simulate the real conformation of the “MT-Loop” and improve the performance of MT1-AF7p, molecular simulations were performed, because this strategy provides multiple methods for predicting the conformation and interaction of proteinase in 3D. In view of the binding site of the receptor–ligand interactions, histidine 4 was selected for mutation to achieve an increased affinity effect. The optimized peptides were further identified and conformed by atomic force microscopy, isothermal titration calorimetry, cell fluorescence imaging *in vitro*, and near-infrared fluorescence tumor optical imaging *in vivo*. The results revealed that the optimized peptide with a mutation of histidine 4 to arginine has the highest affinity and specificity, and exhibited an increased fluorescence intensity in the tumor site in optical imaging.

## Introduction

Matrix metalloproteinases (MMPs) are a family of zinc-dependent endopeptidases that consists of 24 human MMPs, which are capable of remodeling the extracellular matrix (ECM), targeting growth factors, cytokines, cell surface-associated adhesion, and signaling receptors^1–3^. Membrane type 1 MMP (MT1-MMP, also known as MMP14) belongs to the type I transmembrane MMP (MT-MMP) subfamily. From a structural perspective, MT1-MMP is expressed on the cell membrane and contains a potential transmembrane domain. The surface of the extracellular region has a unique segment termed as the “MT-Loop”^4,5^. This area is flexible, exposed, low homological, distant from the catalytic active site, and can be easily assessed by ligands. From a functional perspective, MT1-MMP acts as a collagenase, which is involved in the degradation and remodeling of the extracellular matrix in normal physiological processes, such as embryonic development and reproduction, as well as in diverse aggressive disease processes, such as in lung, liver, oral mucosal, gastric, intestinal, pancreatic, renal, prostate, testicular, colorectal, breast, ovarian, endometrial, cervical, glioma, melanoma, head, and neck cancers^1,2,6–8^. Moreover, MT1-MMP is involved in hyperlipemia, atherosclerosis, influenza-related tissue damage^9^, Alzheimer’s disease^10^, arthritis and Winchester syndrome^11^. Aside from exhibiting pericellular collagenase activity, MT1-MMP activates MMP-2, which is essential for biological and pathological processes, and this activity has been proven to be involved in tumor invasion^3^. An increasing number of studies have reported that MT1-MMP is a promising target for cancer detection and therapy.

Selective receptor-targeting agents are effective tools for targeting or inhibiting specific cells. A ligand that has a specific affinity to MT1-MMP may facilitate the targeting and labeling of malignant tumors that overexpress MT1-MMP. Peptide probes aid in the early diagnosis and effective treatment of tumors. Several studies have demonstrated that polypeptide molecules that can trace MT1-MMP could be used as imaging agents in several cancer models^12–14^. We reported that MT1-AF7p (HWKHLHNTKTFL) was screened and identified by using phage displayed-library that bind to the “MT-Loop” region and was utilized to target the MT1-MMP-overexpressed tumor cells *in vitro* and *in vivo*^15^. Another group employed MT1-AF7p to decorate nanoparticles to mediate tumor targeting, and they obtained the desired effects^16^. Nonetheless, several factors, such as the short half-life time *in vivo*, binding ability, and specificity, could still be improved. Recent studies have been devoted to discovering new bioactive peptides and improving the properties of peptide probes^17^. Chen *et al*. summarized the development of peptide-based imaging agents with emphasis on the probe design^18^. The present work attempts to address these limitations of peptide probes. Computer-aided technology is widely used for designing bioactive peptides, predicting mutation energies to improve performance, and forecasting protein–peptide interactions^19^. Compared with other experimental methods, molecular docking and simulation approaches will decrease the cost of laboratory research since they will be able to perform studies directed to specific predicted protein-peptide interactions^20^. Thus, a molecular dynamics program called GROMACS and a protein–protein molecular docking approach named ZDOCK were used in this study. ZDOCK utilizes fast Fourier transform to perform an exhaustive, grid-based search of the spatial degrees of freedom between two macromolecules^21^.

Here, to obtain a novel peptide with an increased specific affinity and a prolonged half-life time, we used computational methods and rational design to optimize the structure and specificity of MT1-AF7p. Two peptidomimetics with mutation at histidine 4 were obtained and denoted as MT1-AF7p-H4K and MT1-AF7p-H4R. We performed atomic force microscopy (AFM), isothermal titration calorimetry (ITC), and cell fluorescence imaging techniques on the MT1-MMP and 12-residue-optimized peptides *in vitro*. We further showed that compared with the original peptide (MT1-AF7p), MT1-AF7p-H4R had a prolonged half-life time, higher specificity and affinity to the receptor (MT1-MMP) in optical imaging *in vivo*, indicating that molecular simulation is an efficient way of optimizing peptides for cancer detection.

## Methods

### Molecular simulation

#### Preparation of protein

The 3D structure of the MT1-MMP catalytic domain (PDB code 1BQQ) was obtained from the Protein Data Bank (www.rcsb.org). Prior to the docking procedure, the water molecules and the ligand (TIMP-2) were removed from the protein crystal structure by using Discovery Studio (DS)^22,23^ version 2.5. Hydrogen atoms were added by using the CHARMm^24^ force field.

#### Construction of the polypeptide structure

The polypeptide structure (MT1-AF7p) was built by using the Build and Edit Protein module of DS 2.5. Then, a reasonable conformation was obtained by using GROMACS version 4.6.5 with the OPLS-AA/L all-atom force field (2001 amino acid dihedrals). MT1-AF7p was centered in separate cubic boxes and solvated by using the SPC216 water model^25^. The protein had a total charge of 2.000 e; thus, the system needed two CL ions to achieve electroneutrality. Convergence was achieved when a maximum force of less than 1000 kJ mol^−1^nm^−1^ resided on any atom. A two-step equilibration phase was used to independently simulate the constant volume and constant pressure ensembles with 2 ns until the system was well equilibrated at the desired temperature and pressure. Then, molecular dynamics simulations were conducted for 1 μs under the same conditions. The system stability, the differences in the trajectories, and the root mean square deviations were analyzed by using the available tools in the GROMACS package.

The site-directed mutation of MT1-AF7p was executed by using the Build Mutants protocol of DS 2.5, which mutates selected residues to specified types and optimizes the conformation of both the mutated residues and any surrounding residue.

#### Molecular docking of MT1-MMP and the polypeptide

To generate the docking model for MT1-MMP and the polypeptide, molecular docking was conducted by using the Protein Docking module of DS 2.5. The Protein Docking module is a suite of programs for the automatic docking of a protein (or polypeptide) to a receptor, and it uses the classic ZDOCK algorithm to predict complex protein–protein structures. Then, the MT1-AF7p equilibrium conformation was docked into the MT1-MMP model by using ZDOCK^26^ with a 6° angular step size to generate 54,000 poses, of which the top 2000 were re-ranked by ZRANK^27^ and the top 100 were clustered. These poses were subsequently processed with RDOCK^28^, and only clusters with the highest density of poses were further considered.

### Protein expression, purification, and refolding

The MT1-MMP catalytic domain (20 kDa) was expressed in *Escherichia coli* (*E. coli*) as inclusion bodies. The activated form was produced through refold method. The recombinant human MT1-MMP catalytic domain was produced by *E. coli* BL21 that carried expression plasmids. The bacteria were grown to an optical density of 0.4 at 37 °C prior to induction. The overexpression of the protein of interest was induced by adding isopropyl-β-galactoside to a final concentration of 0.5 mM. Cultivation was proceeded for 4 h before the bacteria were broken by an ultrasonic wave. The inclusion bodies (proMT1-MMP) were solubilized and purified through his-tag affinity chromatography (General Electric, USA), as well as refolded by using dialysis bags to gradually reduce high denaturant concentrations in this process. The refolding buffer included 50 mM HEPES, 10 mM CaCl_2_, 200 mM NaCl, 1 mM phenylmethylsulfonyl fluoride, 20 μM ZnCl_2_, 0.01% Brij-35, 5% Glycerol, 100 μg/ml DNase, at a pH 7.5.

### Evaluation of protease activity

The protein samples from induced lysates, inclusion bodies and renaturation were separated by 15% reducing sodium dodecyl sulfate–polyacrylamide gel electrophoresis (SDS–PAGE) gel followed by Coomassie Brilliant Blue R-250 (CBB) staining. Protein concentration was measured at each step by the BCA Protein Assay Kit (Thermo Scientific, USA) according to manufacturer’s instructions. After renaturation of MT1-MMP, the enzymatic activity of the recombinant MT1-MMP was measured by using fluorescein conjugated substrate, DQ™-gelatin from pig skin (Molecular Probes).

### Synthesis and labeling of peptide probes

The peptide probe MT1-AF7p (HWKHLHNTKTFL) was synthesized through solid-phase method by using Fmoc chemistry, and the crude products were purified through reversed phase column chromatography by using LC-8A and SPD-M10A (Shimadzu, Japan) and validated through time-of-flight mass spectrography (AB SCIEX 5800, USA). The polypeptides MT1–160p (REVPYAYIREGHEKQ), MT1-AF7p-H4K (HWKKLHNTKTFL), MT1-AF7p-H4R (HWKRLHNTKTFL), FITC-labeled peptides with purities of 98% were synthesized by GL Biochem Ltd. (Shanghai, China). Cy5.5-labeled peptides with purities of 98% were synthesized and purified in Dr. Lei Zhu’s lab at Xiamen University. In brief, Cy5.5 succinimide easter (Cy5.5-NHS, 1 mg) was coupled to the NH_2_-terminus of MT1-AF7p (4 mg) in 400 μL anhydrous dimethylformamide containing 10% of diisopropylethylamine and shielded from light. The crude peptides were purified and analyzed by HPLC on a C18 column.

### Biomechanical tests

#### Immobilization of MT1-160p or MT1-MMP on substrate

The surface layer of the mica was stripped to expose the silanol group-coated surface. Then, the mica was treated with 3-aminopropyltriethoxysilane (APTES, vapor) for 1.5 h at room temperature and dried in vacuum. To immobilize the protein or the peptide, the activated mica surface was immerged into MT1-160p or MT1-MMP solution (300 µg/mL in PBS) for 15 min, rinsed and kept in PBS buffer.

#### Functionalization of the AFM cantilever tips

AFM cantilever tips (MSCT) were purchased from Veeco (CA). The polypeptides were covalently immobilized on the tips by using chemical reactions as previously described^29^. Briefly, the cleaned AFM tips were immediately transferred to a desiccator flooded with argon, and aminated by incubation with 50 µL APTES and 15 µL *N*, *N*-diisopropylethylamine for 1.5 h through chemical vapor-phase deposition. Then, flexible bifunctional PEG cross-linkers (NHS-PEG18-aldehyde, purchased from Dr. Hermann Gruber’s laboratory, Johannes Kepler University Linz, Austria) were attached to the amino-modified cantilevers by incubating the tips in NHS-PEG18-aldehyde buffer (3.3 mg/mL in chloroform containing 0.5% triethylamine (v/v)) for 2 h at room temperature. Subsequently, the AFM tips were rinsed with chloroform to remove the un-bound NHS-PEG18-aldehyde and dried with argon. The AFM tips were immersed in 100 µg/mL polypeptide solution containing 10 mM NaCNBH_3_ and incubated for 1 h at room temperature. Ethanolamine was added to a final concentration of 25 mM to block the unoccupied aldehyde groups. The prepared AFM tips and samples were cleaned with PBS and stored in the PBS buffer at 4 °C.

#### AFM force curve measurements

The force curves were measured by using a PicoPSM 5500 instrument (Agilent Technologies, MA). The activated mica was placed in a fluid cell, and the polypeptides were covalently immobilized on the AFM cantilever tips. Free excess peptides (1 mmol/L) were added to the dish for the control measurement. The interaction force were detected at different points on the substrate. Over 1,000 force–distance cycles were collected, and a total of 300 force curves were recorded at different positions on the substrate. The average force values were summarized and calculated in the form of histograms and were expressed as a probability (%), which was obtained by dividing each count number by the total number of measured force curves.

### Isothermal Titration Calorimetry

The ITC experiments were conducted by using a MicroCal ITC-200 system (General Electric, USA) to determine the thermodynamic parameters of the polypeptides. The peptide of interest was dissolved in 10 mM HEPES and 150 mM NaCl solution at pH 7.4. All titrations were performed at 25 °C by injecting aliquots of a 40 μL MT1-160p (1 mM) degassed solution in a syringe into the calorimeter cell containing 200 μL of binding peptides (0.1 mM) degassed solution at a stirring rate of 1000 rpm. The baseline was corrected manually and the heat data were processed by fitting the ITC data into the NanoAnalyze software.

### Cell fluorescent labeling by using a confocal laser scanning microscope

MT1-MMP overexpressed tumor cell lines were examined by western blot. In brief, the proteins were extracted from cells, and the protein concentration was measured by BCA Protein Assay Kit (Thermo Scientific, USA). Samples were boiled in Laemmli sample buffer and separated by SDS-PAGE. After transferred to polyvinylidene difluoride (PVDF) membranes, samples were probed with the MT1-MMP antibody or actin, then developed by Pierce ECL 2 western blotting substrate (Thermo Scientific, Rockford, IL). The human fibrosarcoma cell line HT1080 was cultured in RPMI-1640 medium containing 10% (v/v) fetal bovine serum (Invitrogen, USA) supplemented with penicillin (100 μg/mL) and streptomycin (100 μg/mL) at 37 °C under 5% CO_2_. Cells were seeded on a six-well plate with a cover glass at a concentration of 1 × 10^4^ cells/well. After being fixed and blocked on the next day, cells were detected by using fluorophore-labeled peptides at a concentration of 10 nM. The samples were washed thrice at the end of each step. To comparative analyze the affinity between the FITC-MT1-AF7p-H4R and other peptides, the fixed HT1080 cells were incubated with 10 nM peptide ligand and then added with free FITC-MT1-AF7p-H4R to facilitate a competitive reaction. All of the cells were counted with 4′,6-diamidino-2-phenyl indole-containing mounting medium and detected with a confocal laser scanning microscope.

### Whole-body small animal optical imaging

All of the animal experiments were conducted in accordance with the principles and procedures outlined in the ethics committee of Jilin University, and approved by Animal Care and Use Committee (CC/ACUCC) of Xiamen University. Subcutaneous sites of athymic nude mice (BALB/c, 6 weeks old, female, 19–21 g) were injected with a suspension of 1 × 10^7^ breast carcinoma MDA-MB-435 cells in PBS (100 μL). Tumor growth was assessed with caliper measurements every 2 days, and tumor size was determined using the formula: V = a^*^(b^2^)/2, where a is the maximum length and b is the maximum width of each tumor in mm respectively. The tumor-bearing mice (n = 5/group) were subjected to optical imaging experiments once the tumor volume (in the right front leg region) reached average size of 200 mm^3^ (Error less than 18 mm^3^), mice were randomly allocated into three groups: Cy5.5-MT1-AF7p-H4R, Cy5.5-MT1-AF7p-H4R-Block or Cy5.5-MT1-AF7p were injected via their tail vein with 10 μM (1 nM in 100 μL PBS). Fluorescent images and analysis were performed by using an IVIS Lumina II imaging system (Caliper Life Sciences, MA, USA; Excitation Filter: 630 nm, Emission Filter: 700 nm) at different time point post-injection (1, 2, 4, 6, 12, and 24 h). During injection and image acquiring process, mice were anesthetized with 2.5% isoflurane in oxygen at a flow of 1.5 L/min. Images were normalized to the same scale and analyzed. For semi-quantitative comparison, the regions of interest (ROI) were drawn over the tissues of interest, and the scaled average signal (photons cm^−2^ s^−1^) for each area was measured. Fluorescent signals from ROI were further corrected by the mice body weights. Results were presented as the mean ± SD for a group of five animals.

The *ex vivo* imaging of excised tumors and organs further confirmed the targeting specificity of the optimized peptide. The mice were injected with 1 nM Cy5.5-peptide to evaluate the distribution of affinity peptides in tumor tissues and major organs. At 4 h post-injection, the tumor-bearing mice were sacrificed, and their major organs, tissues, and tumors were harvested and placed on a black paper for *ex vivo* imaging (IVIS Lumina II, Caliper Life Sciences, MA, USA). The results were presented as the average scaled signal from the organs and the tumors.

All methods were conducted according to guidelines and regulations of the Key Laboratory of Molecular Enzymology and Enzyme Engineering of the Ministry of Education. All experimental protocols at *in vitro* and *in vivo* level were approved by the department of animal care at Jilin University and Xiamen University.

### Statistical Analysis

Results were exhibited as mean ± SD. Differences within groups and between groups were checked by two-tailed paired and unpaired Student’s t-tests, respectively. ^*^Represent P-value ≤ 0.05, ^**^represent P-value ≤ 0.01, and ^***^represent P-value ≤ 0.001. The results were considered significant with P-values ≤ 0.05.

### Data availability

The authors have declared that the materials, data and protocols in the manuscript are available to readers, and the information for materials is disclosed. The authors declared that experiments on mice were conducted in accordance with the principles and procedures outlined in the ethics committee of Jilin University and Xiamen University.

## Results

### Molecular modeling of MT1-AF7p and MT1-MMP revealed the possible interaction mechanism

**Figure 1:**
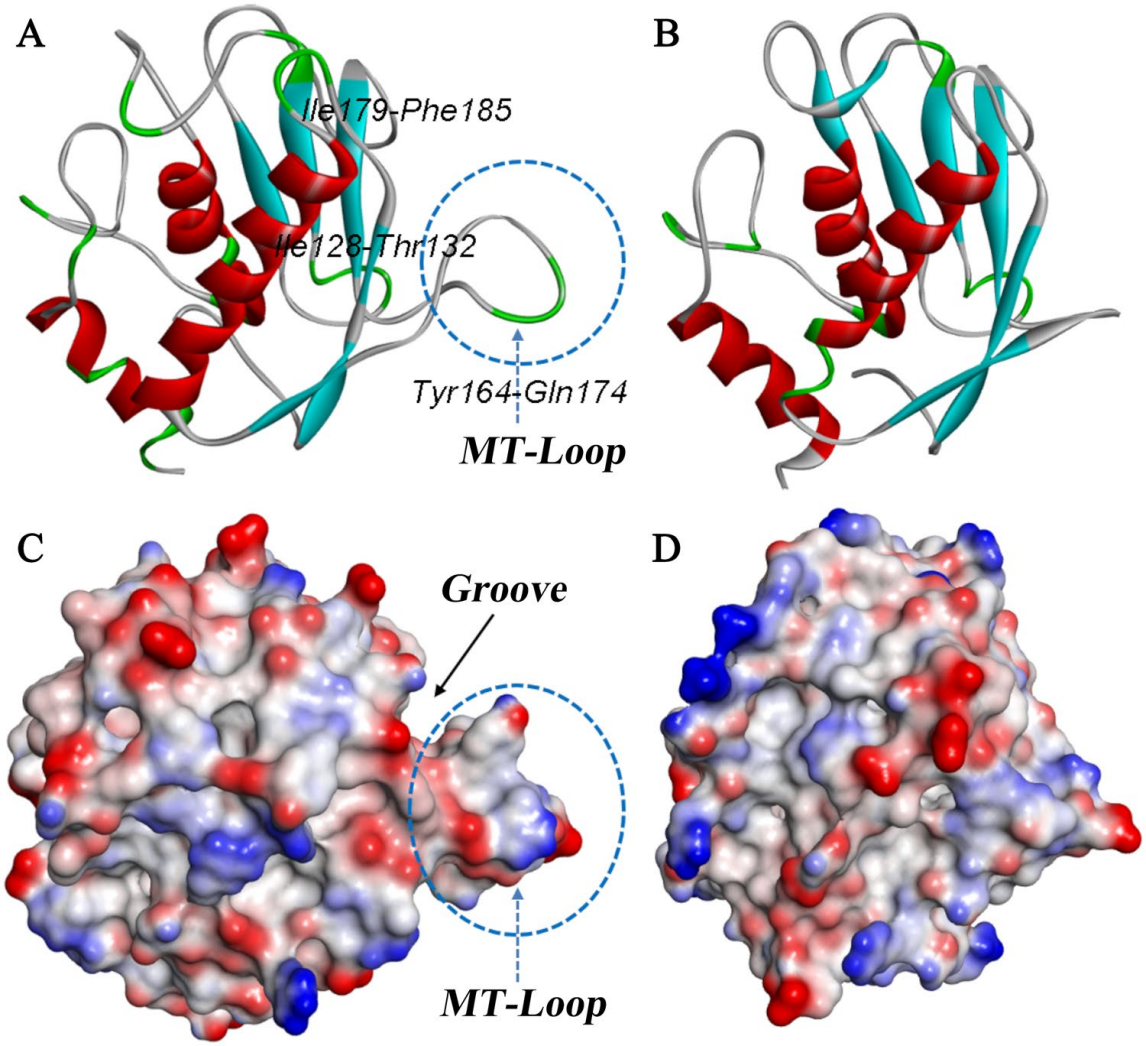
The overall conformation of MT1-MMP and the classical MMP (MMP2). (**A**,**C**) MT1-MMP (PDB code: 1BQQ_M); (**B**,**D**) MMP2 (PDB code: 1QIB_A). Protein (**A**,**B**) are represented by the solid ribbon which are colored by the secondary type; Protein (**C**,**D**) are represented by the surface which are colored by the interpolated charge. Blue represents positive charge and red represents negative charge. The conformation in the dotted circle is MT-Loop. An arrow is pointed at the groove.

MT1-AF7p, which exhibited a binding ability to MT1-MMP in the physical studies and in the imaging at the cellular and organism levels^15,30^. Here, to optimize the affinity and specificity of MT1-AF7p and characterize their interactions, computer-aided methods, including molecular modeling and docking experiments were employed. The conformation of the MT1-MMP catalytic domain had identical regular secondary structure elements as that of classical MMPs: three α-helices and five stranded β-sheets (Fig. 1A,B). The most significant deviation occurred in Tyr164-Gln174, where the chain bulged out to form a “MT-Loop”^4^. In contrast to classical MMPs, the typical MT-Loop in MT-MMP, established a groove with Ile128-Thr132 and Ile179-Phe185 (Fig. 1C,D).

Molecular interactions between peptides and MT1-MMP mediate many biological and pathological processes. To study the possible interaction mechanism, MT1-AF7p was docked into MT1-MMP by using the ZDOCK module of DS 2.5. The optimal structure of MT1-AF7p was the average conformation in the last 100 ns after 1 μs of molecular dynamics simulations (Fig. S1). As shown in Fig. S2, the top poses were located in the largest cluster (the top 100 poses from ZDOCK were re-ranked by ZRANK and clustered) were mostly processed around the groove near the MT-Loop. All of the top poses were then evaluated with RDOCK, and the cluster with the highest density of poses was considered. The best pose on the basis of the lowest RDOCK energy (−62.87 kcal/mol) (Table S1) was kept for each conformation (Fig. S3A). A detailed analysis of the amino acids of MT1-MMP that bonded to MT1-AF7p in shown in Fig. S3B. Tyr166, Phe181, Glu183, and Asp212 could form six hydrogen bonds with His6, Asn7, Thr8, Lys9, and Leu12 of MT1-AF7p (Table S2). As shown in Fig. S3B, the MT1-AF7p residues within 7 Å of any residue of MT1-MMP were His4, Leu5, and Phe11, all of which could be mutated to achieve a greater affinity effect aside from the residues that formed hydrogen bonds with MT1-MMP. By contrast, the other residues of MT1-AF7p, namely, His1, Trp2, Lys3, and Thr10, were not in the range of 7 Å of any residue of MT1-MMP. His1, Trp2, and Lys3, were at the N terminal of MT1-AF7p, suggesting that they be could useful biomarkers even though they are far away from MT1-MMP.

### Optimization of MT1-AF7p

**Figure 2:**
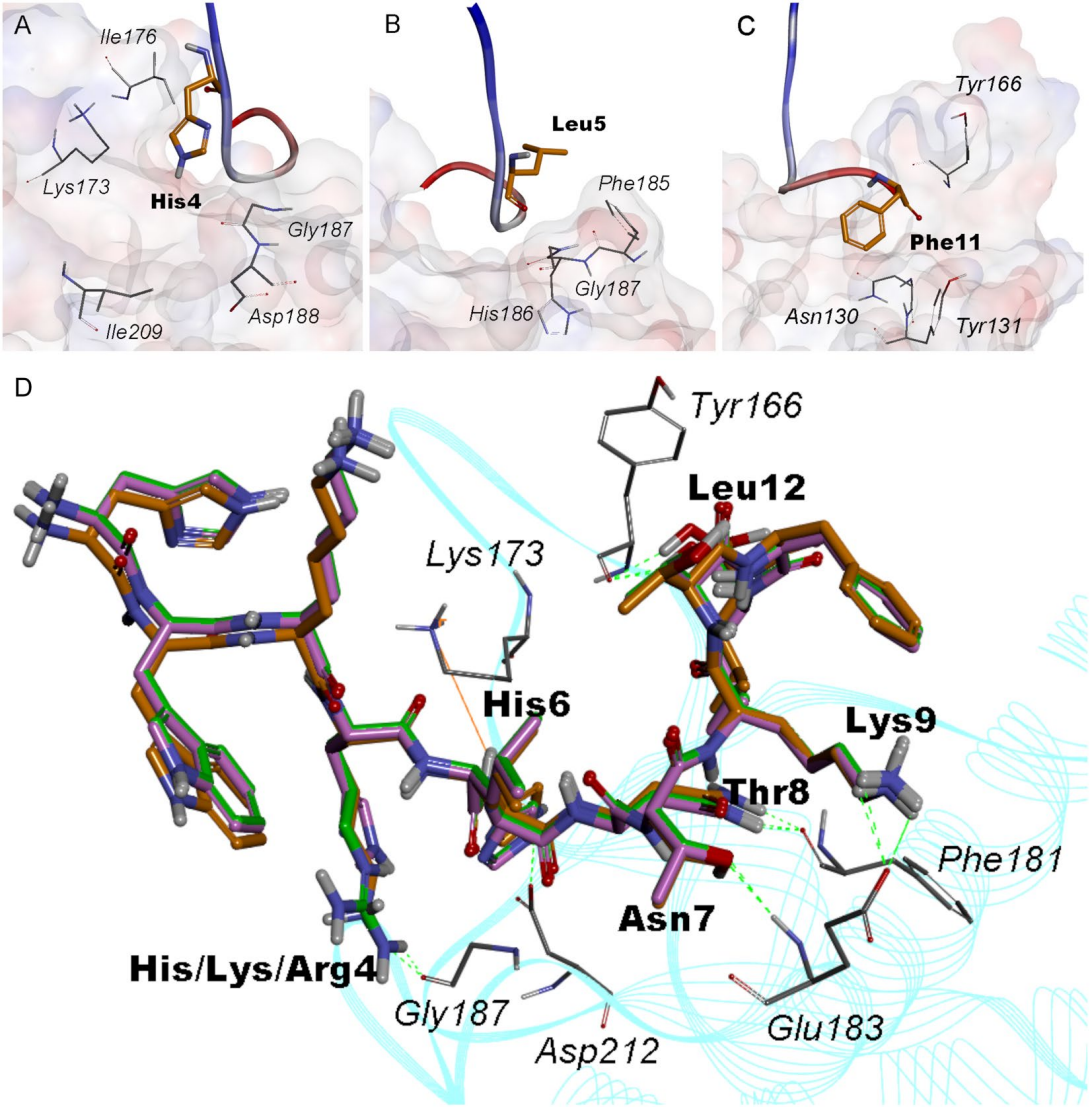
The schematic of the amino acid MT1-MMP around the amino acid to be mutated of MT1-AF7p at a 7 Å radius and the interrelation between affinity peptides superposition. The amino acid to be mutated of MT1-AF7p was (**A**), His4; (**B**), Leu5; (**C**), Phe11. In Figures (**A**–**C**), the amino acid of MT1-AF7p is displayed in the stick model, whose carbon skeleton is painted orange. MT1-AF7p is represented by a solid ribbon colored by the N-C terminal. The amino acids of MT1-MMP around the 7 Å radius are shown by the line, and the carbon skeleton of the amino acid of MT1-MMP is painted grey. Figure D shows the superposition between MT1-MMP and MT1-AF7p/MT1-AF7p-H4K/MT1-AF7p-H4R. In figure D, MT1-MMP is represented by the cyan line ribbon. The amino acid of MT1-MMP that formed hydrogen bonds are displayed in line model. The carbon skeleton of the amino acid of MT1-MMP was painted grey. The MT1-AF7p/MT1-AF7p-H4K/MT1-AF7p-H4R superposition are displayed in stick model representation with different colors of carbon skeletons: MT1-AF7p, magenta; MT1-AF7p-H4K, green; and MT1-AF7p-H4R, orange.

Previous studies showed that the “MT-Loop” favors MT1-AF7p residues at the groove positions of MT1-MMP^31^. His6, Asn7, Thr8, Lys9, and Leu12 of MT1-AF7p form hydrogen bonds with MT1-MMP. Thus, to improve the binding ability of MT1-AF7p to MT1-MMP, His4, Leu5, and Phe11 were mutated. In Fig. 2A, Lys173, Ile176, Gly187, Asp188, and Ile209 of MT1-MMP were selected within 7 Å of His4 of MT1-AF7p. Therefore, if His4 mutated into Lys or Arg, depending on which basic amino acid had a longer chain, His4 might form hydrogen bonds with the carbonyl oxygen of Gly187. By contrast, Phe185, His186, and Gly187 of MT1-MMP were in the range of 7 Å of Leu5 of MT1-AF7p, and Phe185 was closer to the Leu5 side chain (Fig. 2B). Leu5 is not going to mutate to any amino, because both Phe185 and Leu5 are hydrophobic amino acids that could form a hydrophobic interaction together. Similarly, Phe11, which could also produce a strong hydrophobic interaction with Tyr131 and Tyr166 (Fig. 2C), was also retained. In summary, two mutations were identified: MT1-AF7p-H4K and MT1-AF7p-H4R.

To confirm that the mutations had an optimal affinity to MT1-MMP, we docked the two polypeptides to MT1-MMP by using the aforementioned method and calculated the interaction energy of the polypeptide/MT1-MMP complexes. As shown in Fig. 2D, MT1-AF7p-H4K and MT1-AF7p-H4R were tightly bound to nearly the same site as MT1-AF7p, in the groove of MT1-MMP. Table S1 lists the RDOCK scores (lower scores are better). The RDOCK score of MT1-AF7p-H4K and MT1-AF7p-H4R were −65.11 and −68.99 kcal/mol, both of which were lower than that of MT1-AF7, implying that the mutations of MT1-AF7p presented a more optimal binding. The hydrogen bonds formed between the polypeptides and MT1-MMP contributed to the stability of the complexes, and a greater number of hydrogen bonds resulted in a more stable complex. Tables S3 and S4 show the detailed parameters of the hydrogen bond between the polypeptides and MT1-MMP. When His4 mutated into Lys or Arg, the H of the extended side chain formed hydrogen bonds with the carbonyl oxygen of Gly187 of MT1-MMP (Fig. 2D). His6 of MT1-AF7p-H4K lost the hydrogen bonds but formed a pi–pi interaction with Asp212 of MT1-MMP (Table S5). In conclusion, the mutations could form an additional non-bond interaction with MT1-MMP than with MT1-AF-7p. MT1-AF7p-H4K formed an additional pi–pi interaction, and MT1-AF7p-H4R formed an additional hydrogen bond. Thus, on the basis of the docking results, the polypeptides that mutated with a more favorable interaction energy were selected for further analysis.

### AFM analysis of the intermolecular force between MT1-MMP and peptidomimetics

**Figure 3:**
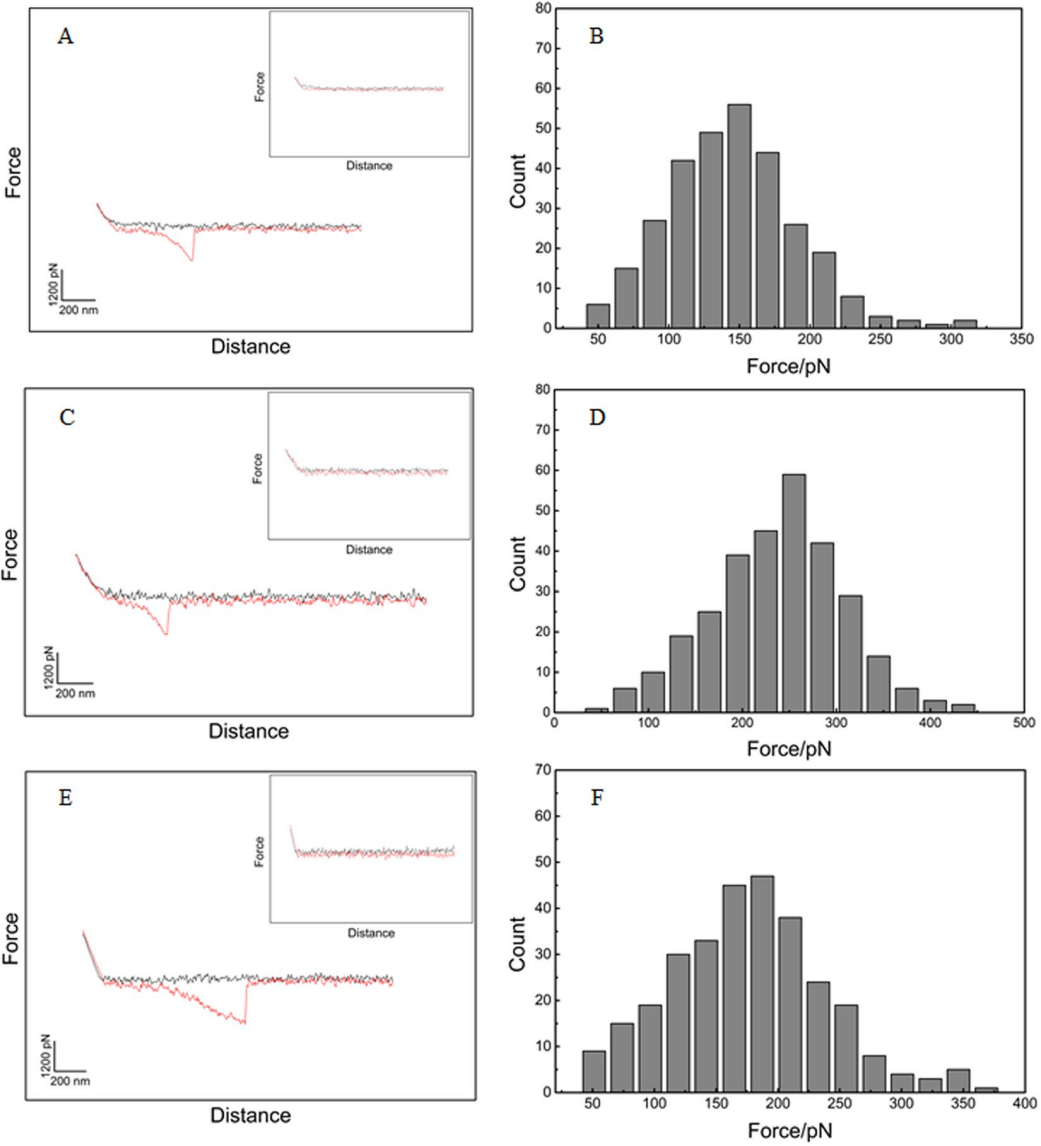
The force measurement of optimized peptides and MT1-MMP. These typical force curves showing the interactions between MT1-AF7p and MT1-MMP (**A**), MT1-AF7p-H4R and MT1-MMP (**B**) or MT1-AF7p-H4K and MT1-MMP (**C**) at 25 °C. An AFM tip functionalized with polypeptide approaches to the substrate where MT1-MMP is immobilized, as shown in panels A,C and D, in the trace process, the deflection shows zero when cantilever is not bent, as the tip comes into contact with substrate, the complexes were formed and the cantilever bends upwards. In the retrace process, the AFM cantilever has an opposite direction to the MT1-MMP coated surface, which result in a binding force compared with the trace process, then the cantilever tethered with polypeptide relaxes and unbends until the repulsive force drops to zero. As a control experiment, excessive free peptides added into reaction cell, the cantilever jumps back to zero deflection (inside). The average force values were expressed as a probability in the form of histograms (panels B, D and F).

AFM was conducted in the following chemical reaction experiment to evaluate the specific interaction forces between each optimized peptide and MT1-MMP. MT1-MMP was expressed in *E.coli* as inclusion bodies and re-natured with catalytic activity (Fig. S4), MT1-MMP showed high enzymatic activity (9.81 × 10^6^) against DQ-gelatin substrate. The optimized peptide was covalently conjugated onto an APTES-coated AFM tip via a flexible heterobifunctional polyethylene glycol cross-linker (NHS-PEG18-aldehyde). The NHS-ester of the linker bound to the APTES-coated tip through the NHS-ester end, and the free aldehyde group end was designed to covalently bind the protein or the peptide. The characteristic parabolic bending curve was caused by the stretching of the PEG linker. Figure 3 shows a typical force–distance cycle with a single-molecule recognition event that demonstrates the retraction forces between peptides. The negative force indicates an attraction between MT1-MMP and the peptide probes (MT1-AF7p (3A), MT1-AF7p-H4R (3C), and MT1-AF7p-H4K (3E)). By contrast, a control experiment was performed by adding a free blocking reagent (free excess peptide) to the fluid cell of the MT1-MMP-immobilized substrate, and no peak was revealed (inset). To estimate the strength of the specific affinity, the averaged retraction force was determined for each force curve of the peptide probe against MT1-MMP. We then summarized and analyzed these forces in the form of histograms that fit well to Gaussian curves (Fig. 3B,D,F). As shown in Fig. 3C,D, MT1-AF7p-H4R had the highest affinity to MT1-MMP. The peak position of the histogram was at 260 pN, which had the lowest interaction energy base on its energy rank ordering. This finding agreed with the molecular docking results. MT1-AF7p-H4K (Fig. 3E,F) had a nearly identical binding capacity with MT1-MMP than MT1-AF7P (Fig. 3A,B) at 166 and 150 pN, respectively. Similarly, the force curve between MT1-160p and the optimized peptides showed the same trend (Fig. S5). The AFM single-molecule recognition force spectroscopy (SMRFS) results showed that MT1-AF7p-H4R were tightly bound to its target, MT1-MMP, as predicted by the molecular modeling data, whereas the mutated peptide MT1-AF7p-H4K showed opposite trend.

### Peptidomimetics binds to MT1-MMP in ITC binding assay

**Figure 4:**
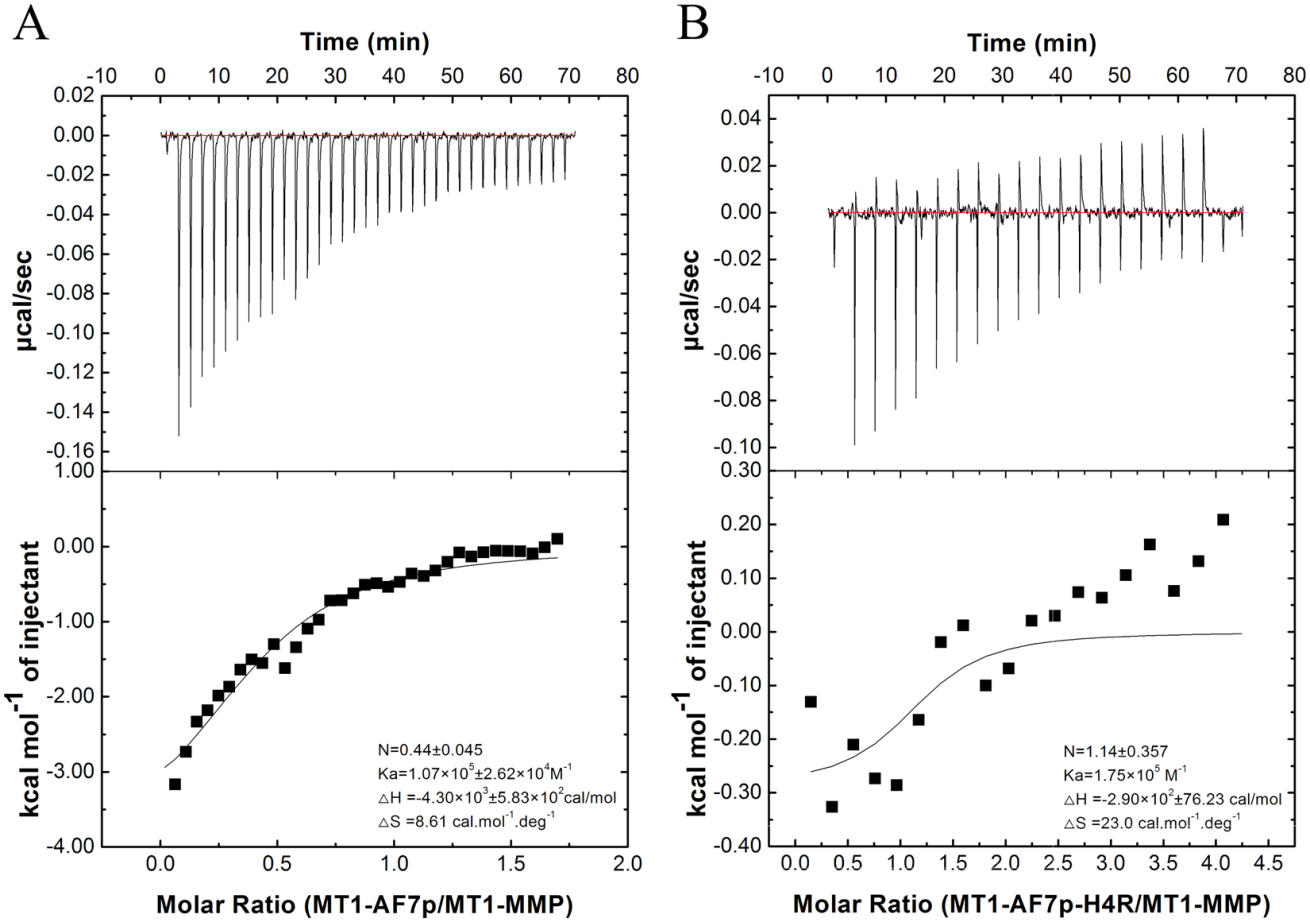
Typical ITC data for injections of optimized peptides into a solution of MT1-MMP protein at 25 °C. The binding isotherms of MT1-AF7p (**A**) and MT1-AF7p-H4R (**B**) were fitted through a simple two-state binding model. Ka, ∆H and ∆S were shown inside the figures. Molar ratio denoted the heat of reaction of optimized peptides and MT1-MMP, the solid line denoted the best fits to experimental data. The binding isotherms were fitted by using the MicroCal Origin package, and the best curve fitting was obtained by using a one-site binding model.

To further validate and characterize the interaction of MT1-MMP with its optimized 12-residue peptides, we measured the interaction by using ITC to titrate MT1-AF7p and MT1-AF7p-H4R into MT1-MMP. Figure 4 shows the typical thermodynamic titration curves of the binding affinity between MT1-MMP and the polypeptides. An exothermic binding reaction was observed as illustrated, and the corresponding thermodynamic parameters are presented inside the figures. The thermodynamic titration curves decreased as the number of injections increased and the concentration of free peptides in the cell decreased simultaneously. Figure 4A and B showed a spontaneous reaction, the negative enthalpy (ΔH < 0) and positive entropy (ΔS > 0) values indicated that hydrophobic forces and electrostatic interactions played pivotal roles in the binding process. MT1-AF7p-H4R showed that it is an enthalpy-driven process and exhibited better thermodynamic parameters (ΔH = −2.9 × 10^2^ cal/mol, ΔS = 23 cal/mol/deg, and the stoichiometry N = 1.14). The binding constant (*K*a) of the affinity peptide MT1-AF7p-H4R (1.75 × 10^5^ M^−1^) to MT1-MMP was higher than that of MT1-AF7P (1.07 × 10^5^ M^−1^).

### Staining of cells with optimized probes

**Figure 5:**
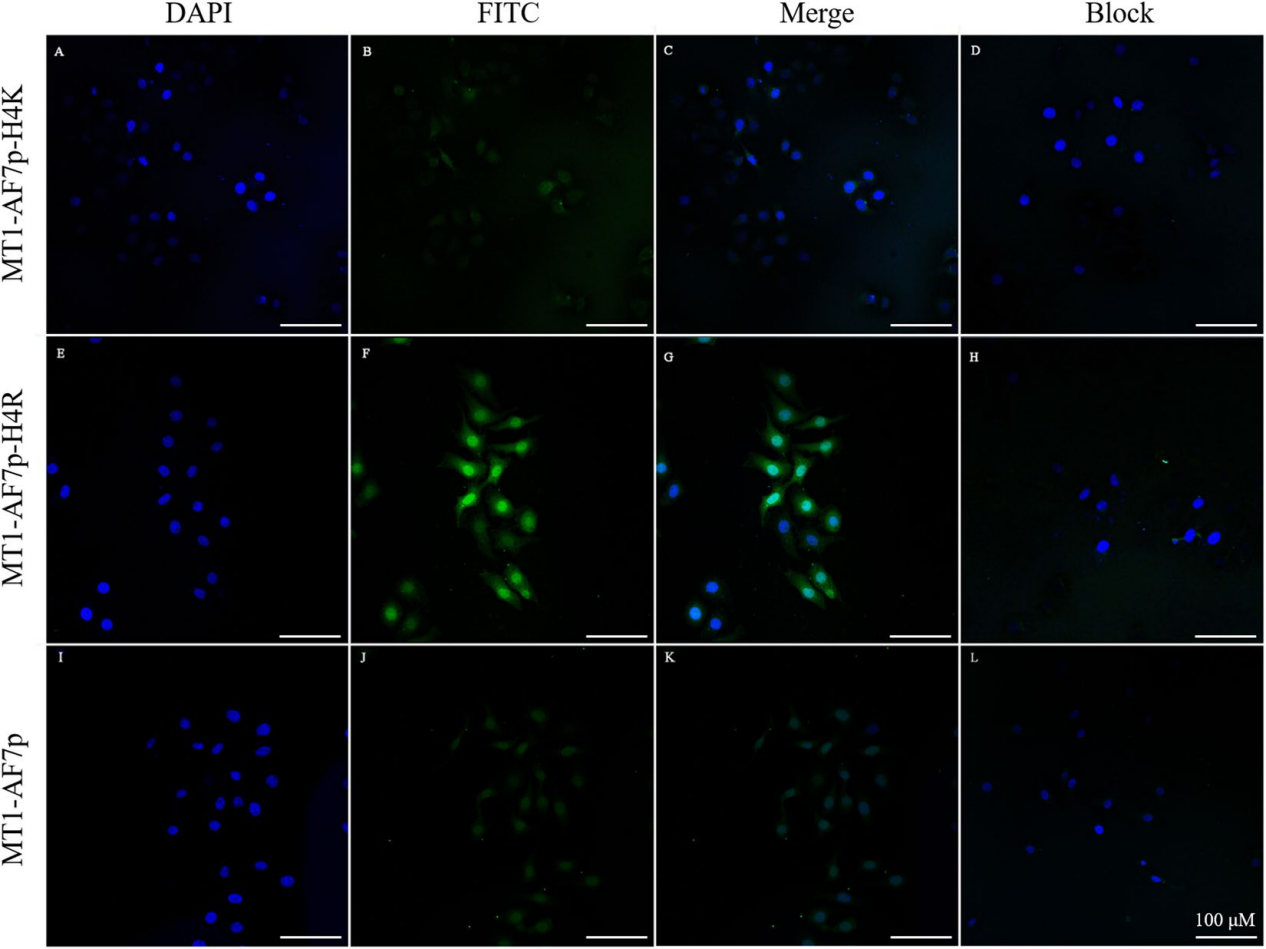
Fluorescence imaging capability of polypeptides. Confocal fluorescence images of HT1080 cells incubated with FITC (Green) labeled polypeptide ligand FITC-MT1-AF7p-H4K, FITC-MT1-AF7p-H4R and FITC-MT1-AF7p for cell surface imaging. DAPI (blue) were used for staining of nucleus. Overlapping images are shown in the merge group.

Given that optimized peptides can target and label MT1-MMP in biophysical experiments, we performed cell fluorescence imaging experiments to examine the binding ability of the optimized peptides at the cellular level *in vitro*, as a previous study showed that MT-MMPs were expressed differentially in tumor models^32^. The protein expression of MT1-MMP was examined by western blot. As Fig. S6 shown, human fibrosarcoma HT1080 and human breast cancer cell line MDA-MB435 are MT1-MMP positive model, in which have higher expression of MT1-MMP than in A549. Thus, in this section, cellular fluorescence was detected in the HT1080 cells. Under culture conditions, MT1-AF7p-H4R exhibited a better imaging ability than MT1-AF7p-H4K and the original polypeptide MT1-AF7p (Fig. 5). To test the binding specificity, free polypeptides were added into the culture wells. The fluorescence signal disappeared because of the competition (Fig. 5), suggesting that MT1-AF7p-H4R, which was predicted and designed through molecular modeling methods, was specific enough to bind to MT1-MMP.

### *In vivo* imaging of MT1-MMP using Cy5.5-labeled optimized peptides

Among the mutated peptides selected by virtual screening and docking based on the calculated lowest binding energy, the binding affinities were tested by conducting AFM, ITC, and cell fluorescence imaging *in vitro*. The tumor targeting effects of the optimal peptide were further confirmed by performing an *in vivo* imaging experiment in a tumor-bearing mice model. The immunohistochemistry and histological examination have proved higher expression of MT1-MMP in breast cancer cell line MDA-MB-435 tumor. Furthermore, MDA-MB-435 xenografts had significantly higher tumor accumulation of Cy5.5-MT1-AF7p than that in negative control^15^. Thus, we seeded MDA-MB-435 tumor cells in mice and evaluated the specificity and affinity of the peptides to MT1-MMP *in vivo*. Free Cy5.5 dye was spread in the whole body and did not accumulate in the tumor site within 48 h post-injection, it demonstrated that the effect of passive targeting capabilities of the Cy5.5 dye was not significant^33^. The fluorescent label Cy5.5 was conjugated onto the N-terminate of MT1-AF7p and MT1-AF7p-H4R. After the Cy5.5 labeled peptides were injected through the tail vein, the time-dependent biodistribution of the peptides were observed by performing NIRF imaging on the live animals. The fluorescent signals detected in the MDA-MB-435 tumors in Fig. S7, indicating that the imaging effect of MT1-AF7p-H4R was better than MT1-AF7p (p ≤ 0.01). The intensity around the tumors disappeared when free polypeptides were injected to block the peptides bound to MT1-MMP. The fluorescence intensity of MT1-AF7p-H4R in the tumor site was considerably stronger than that of MT1-AF7p at all the time points from 2 h to 24 h.

**Figure 6:**
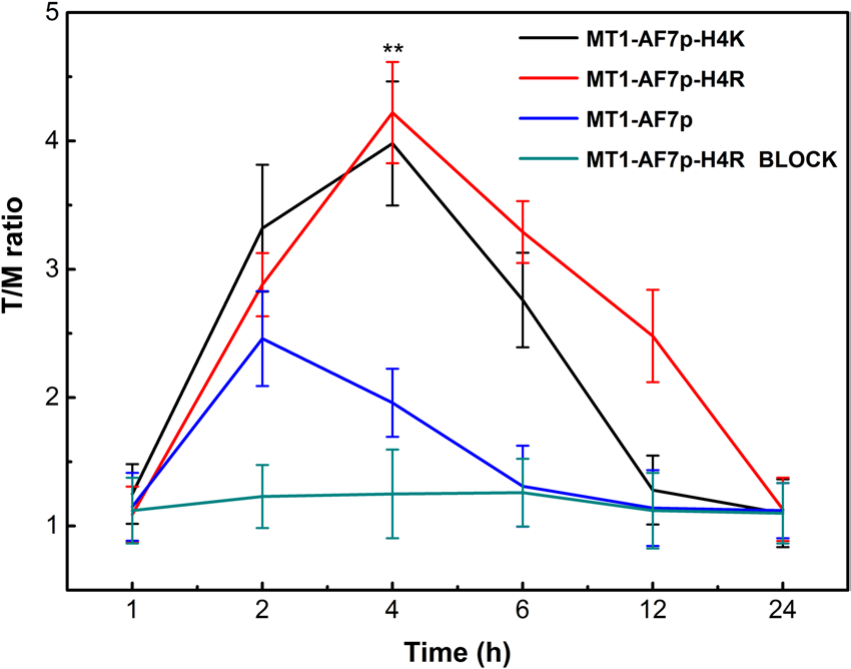
Tumor/muscle (T/M) ratio showed the accumulating ability of peptide probes in post-injection at tumor sites. There was a significant difference between the MT1-AF7p-H4R and MT1-AF7p treatment, ^**^Represents P ≤ 0.01.

**Figure 7:**
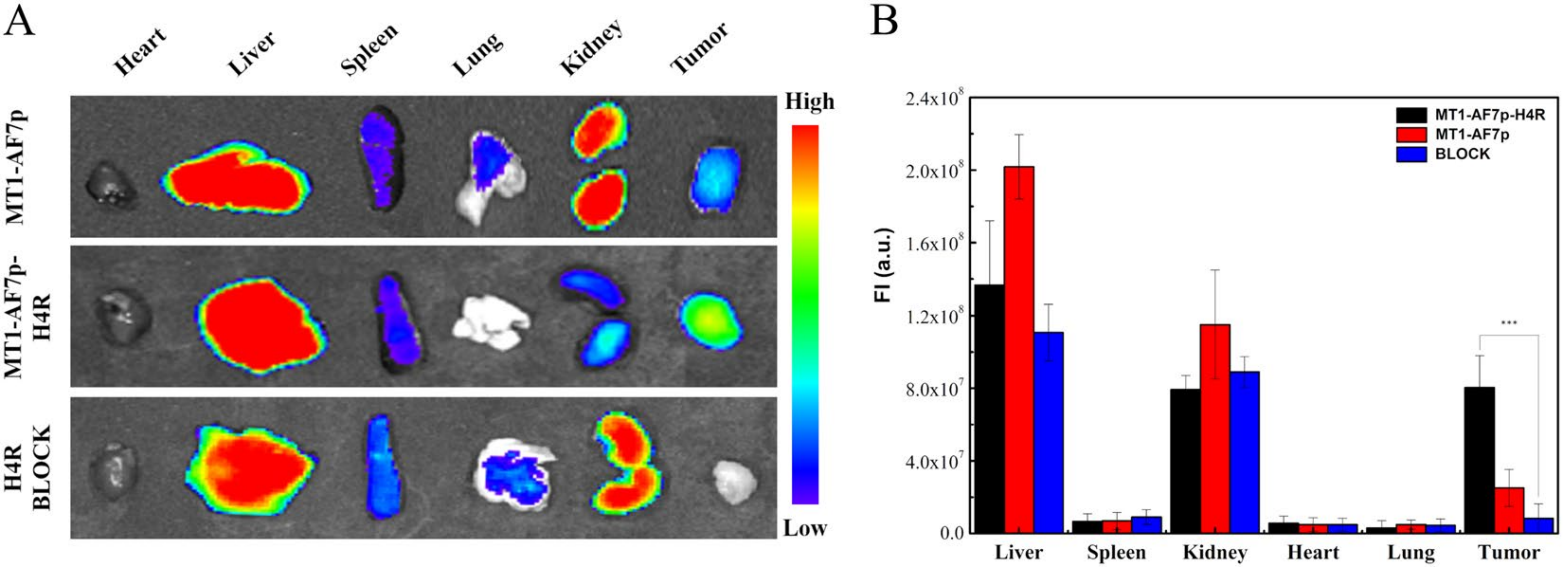
Biodistribution of the peptide ligands *in vivo*. (**A**) *Ex vivo* images of dissected organs of mice bearing breast tumor sacrificed 4 h after intravenous injection of MT1-MMP affinity peptides. (**B**) Analysis of the fluorescent intensity of the formulations in different organs and tumor. Significant fluorescent signals were observed in tumor due to Cy5.5-MT1-AF7p-H4R specific accumulation. The colored bars represent mean values and the error bars represent standard deviation. ^***^Represents P ≤ 0.001.

The tumor-to-normal area ratios are shown in Fig. 6, Cy5.5-MT1-AF7p-H4R accumulates in MDA-MB-435 tumor with time (Tumor/ Muscle ratio: 1.22 ± 0.22 at 1 h p.i. and 2.77 ± 0.31 at 2 h p.i.) and reaches its maximum at 4 h p.i. (T/M: 4.26 ± 0.56). After that, the fluorescent signal starts to decrease by reason of the metabolism. To confirm the binding specificity of Cy5.5-MT1-AF7p-H4R to MT1-MMP, an excess of free MT1-AF7p-H4R (1 μM) was injected 30 min prior to Cy5.5-MT1-AF7p-H4R. Low Cy5.5-MT1-AF7p-H4R accumulation was observed because the MT1-MMP were blocked with excessed free MT1-AF7p-H4R. Following the same treatment, the tissue distribution was assessed in mice. We performed *ex vivo* optical imaging at the experimental endpoint (Fig. 7A). As shown in Fig. 7B, the fluorescence intensities were calculated from organs and tissues, such as the heart, the liver, the spleen, the lung, the kidney, and the tumor. A significantly higher accumulation level of Cy5.5-MT1-AF7p-H4R was observed in the tumor (p-value ≤ 0.001). As expected, compared with MT1-AF7p, the fluorescent signals of Cy5.5-MT1-AF7p-H4R were 3.2 times higher (8.04 × 10^7^ *vs*. 2.51 × 10^7^) were observed, and very few fluorescent signals were detected in the blocking group. Furthermore, the *ex vivo* quantification corroborated the observations from the *in vivo* optical imaging ROI analysis, which exhibited that Cy5.5-MT1-AF7p-H4R had a prominent renal clearance. Therefore, Cy5.5-MT1-AF7p-H4R exhibited a better specific and binding affinity to MT1-MMP than Cy5.5-MT1-AF7p in tumors.

## Discussion

Compared with other micromolecules and antibodies, the peptide probe displayed a better biosafety and a number of advantages, such as lower production cost and better permeability and functionalization at nanomolar concentrations^16,17^. Furthermore, the kidney plays a key role in eliminating drug carriers from the body^18^. A smaller size and molecular weight of the peptide for is more favorable for glomerular filtration^16–18^. In addition, through computer-aided techniques, traditional phage display method has moved from 2D to 3D in the screening of the affinity of peptides. Rational optimization significantly enhanced the specificity and affinity of peptide probe *in vitro* and *in vivo*. MT1-AF7p, which exhibited a binding ability to MT1-MMP in the physical studies and in the imaging at the cellular and organism levels, was employed to decorate the nanoparticles to kill glioma cells^15,30^. Considering that the specificity and binding force of MT1-AF7p were weaker than that of the antibody to MT1-MMP, we posited that the mutagenesis of key amino acid residues could optimize the binding ability of MT1-AF7p. Computer-aided approaches provided tools for binding force enhancement and were suitable for the directly predicting specific ligand–receptor interactions and structures^20,34^. Here, to optimize the affinity and specificity of MT1-AF7p and characterize their interactions, computer-aided methods, including molecular modeling and docking experiments, were employed. We analyzed and predicted the polypeptide structures, the contributions of each amino acid, and the possible interaction mechanism. The results revealed that most of the peptide mutations with optimal scoring, ranking, and docking power bound to the “groove” formed by the “MT-Loop” and nearby amino acid sequences (Fig. 1). A systemic virtual mutation of the key amino acids of MT1-AF7p was performed to optimize its amino acid sequence for an increased binding affinity to MT1-MMP. We found that the mutation of the original ligand (MT1-AF7p) at His4 would optimize the conformation, energy, and chemical bonds in peptidomimetics and MT1-MMP complexes (Fig. 2).

Powerful biophysical approaches, such as AFM^35^ and ITC^31,36^ employed binding force detection methods to identify the receptor–ligand interaction in a single molecule and the energy level because of their accuracy in binding force detection^37–39^. AFM also provides information on the binding mechanisms, dynamics of recognition processes, and interaction-energy landscapes between the interacting biomolecular pairs^40^. ITC measures the generation or consumption of heat following the titration of a ligand onto a protein (or vice versa)^41^. The ability to detect or predict both the binding affinity and the specificity remain limited in methodology^36^. Therefore, this study introduced the combination of multi-test methods to improve the accuracy of binding force detection. Our results suggested that peptidomimetics have a more favorable specific binding force (Figs 3 and 4). The cell labeling (Fig. 5) and optical imaging (Figs 6 and 7) experiments demonstrated a specific targeting of peptidomimetics to MT1-MMP-expressing cells and tumors in a key amino acid-dependent manner, thereby contributing nearly the entire binding force. For the clinic applicable purpose, the more precise quantitative data on pharmacokinetics will require radiolabeled MT1-AF7p-H4R in positron emission tomography (PET) and single-photon emission (SPECT) in a subsequent study.

This study is focused on investigating the specific protein–peptide interactions between the ligand–receptor proteins to try to gain insight into the peptide affinity to MT1-MMP and the critical binding amino acid residues to provide a vehicle for near-infrared fluorescence tumor optical imaging *in vivo*. A novel peptide MT1-AF7p-H4R (HWKHLHNTKTFL) is an ideal bioprobe for the diagnosis and intraoperative fluoroscopic imaging of human malignant tumors. The findings suggested that the application of computer-aided techniques may be a useful approach in the design and development of tumor targeting, detecting, and inhibiting peptide ligands.

## Electronic supplementary material

Supplementary Information
