# Supplementary material for: Optimization of a MT1-MMP-targeting Peptide and Its Application in Near-infrared Fluorescence Tumor Imaging
Source: Sci Rep. 2018 Jul 9;8:10334. doi: 10.1038/s41598-018-28493-9 (PMC6037669; doi:10.1038/s41598-018-28493-9)
Supplement: Supplementary file 1 — Supplementary Information [file 41598_2018_28493_MOESM1_ESM.docx]

**Supporting Information**

***for***

**Optimization of a MT1-MMP-targeting Peptide and Its Application in [Near-infrared Fluorescence Tumor Imaging](https://www.ncbi.nlm.nih.gov/pubmed/22335547)**

*Li Ren^†a,b^, Ye Wang^c†^, Lei Zhu^d^, Liqiao Shen^e^, Jinrui Zhang^e^, Jingjing Wang^f^, Haolong Li^e^, Qingchuan Zheng^g^, Dahai Yu^e^, Xuexun Fang^e*^.*

*† There authors contribute equally.*

** To whom correspondence should be addressed.*

*E-mail: fangxx@jlu.edu.cn.*

*a. College of Food Science and Engineering, Jilin University, 5333 Xi’an Street, Changchun, Jilin 130062, P. R. China.*

*b. State Key Laboratory of Inorganic Synthesis and Preparative Chemistry, Jilin University, 2699 Qianjin Street, Changchun 130012, P. R. China.*

*c. School of Life Science, Jilin University, 2699 Qianjin Street, Changchun, Jilin 130012, PR China.*

*d. Department of Surgery, Emory University School of Medicine, 201 Dowman Drive, Atlanta, GA 30322, United States.*

*e. Key Laboratory of Molecular Enzymology and Enzyme Engineering of the Ministry of Education, Jilin University, 2699 Qianjin Street, Changchun, Jilin 130012, P. R. China.*

*f. State Key Laboratory of Molecular Vaccinology and Molecular Diagnostics & Center for Molecular Imaging and Translational Medicine, Xiamen University, Siming South Road, Xiamen, Fujian 361005, P. R. China.*

*g. Laboratory of Theoretical and Computational Chemistry, Jilin University, Jiefang Road, Changchun, Jilin 130023, P. R. China.*

**Corresponding Author**

fangxx@jlu.edu.cn


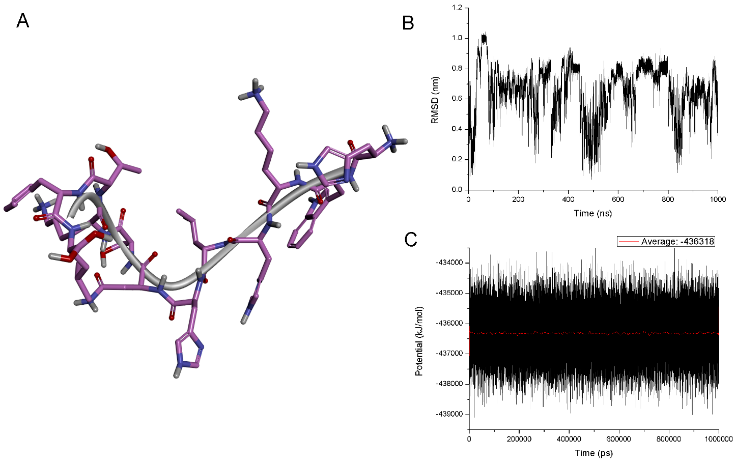


**Fig. S1.** The results of MT1-AF7p during 1 μs MD simulation. A, the average conformation.

B: RMSD plot. C: potential energy.


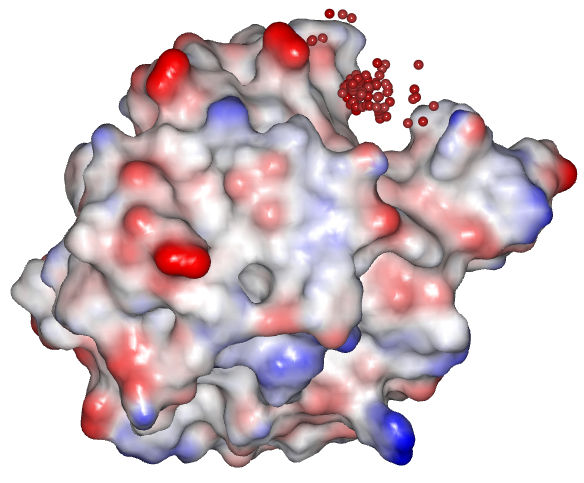


**Fig. S2.** Docking results between the crystal structure of MT1-MMP and MT1-AF7p. Localization of the top 100 poses in the largest cluster are shown by red ball. MT1-MMP is shown by its molecular surface that colored by interpolated charges.


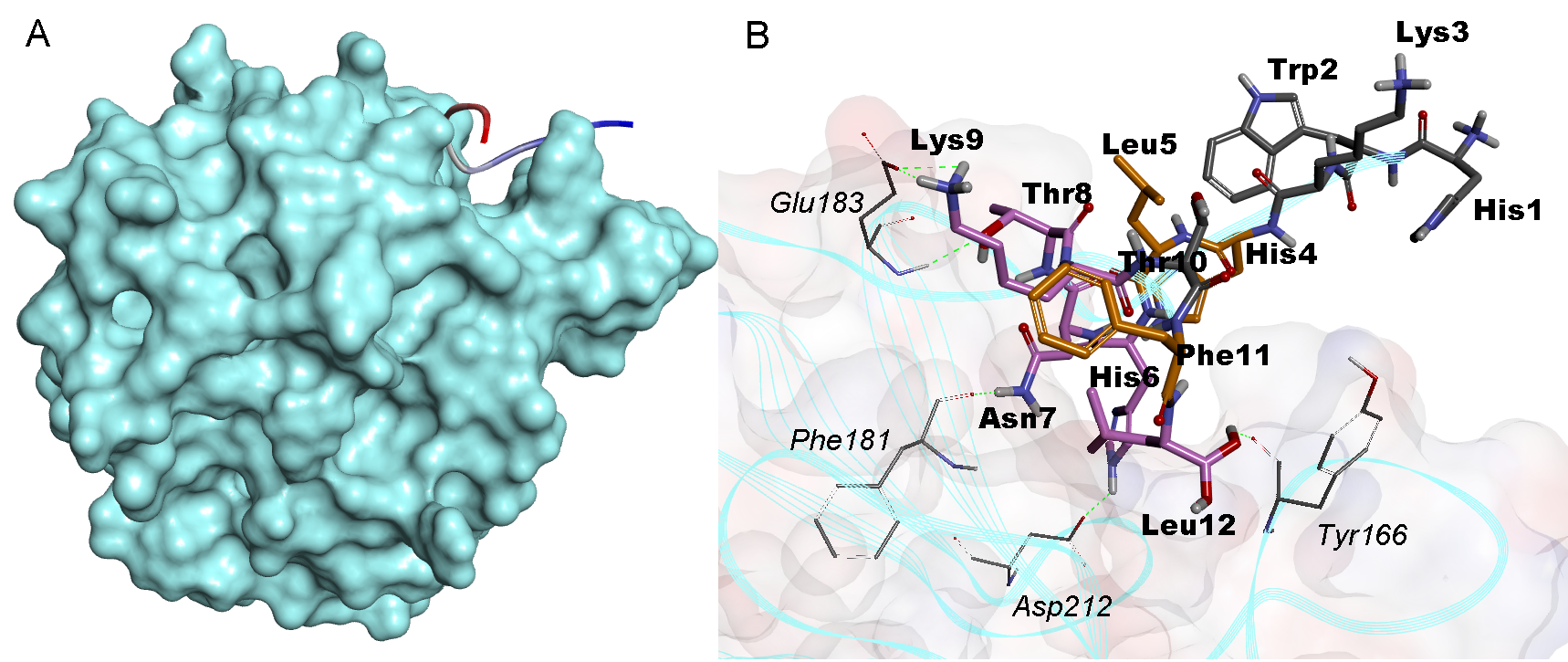


**Figure S3.** Fig A shows the schematic of interrelation between MT1-AF7p and MT1-MMP. MT1-MMP is represented by the cyan surface; MT1-AF7p is represented by a solid ribbon colored by the N-C terminal. Fig B shows the detail of hydrogen-bond interactions between MT1-AF7p and MT1-MMP. The amino acid of MT1-MMP which that formed hydrogen bonds are displayed in line model. The carbon skeleton of the amino acid of MT1-MMP was painted grey. However, the amino acid of MT1-AF7p are displayed in stick model representation with different colors of carbon skeletons: magenta (which has hydrogen-bond interactions with MT1-MMP), orange (prepare to be mutated), grey (away from MT1-MMP). Hydrogen bonds are represented by the green dashed.


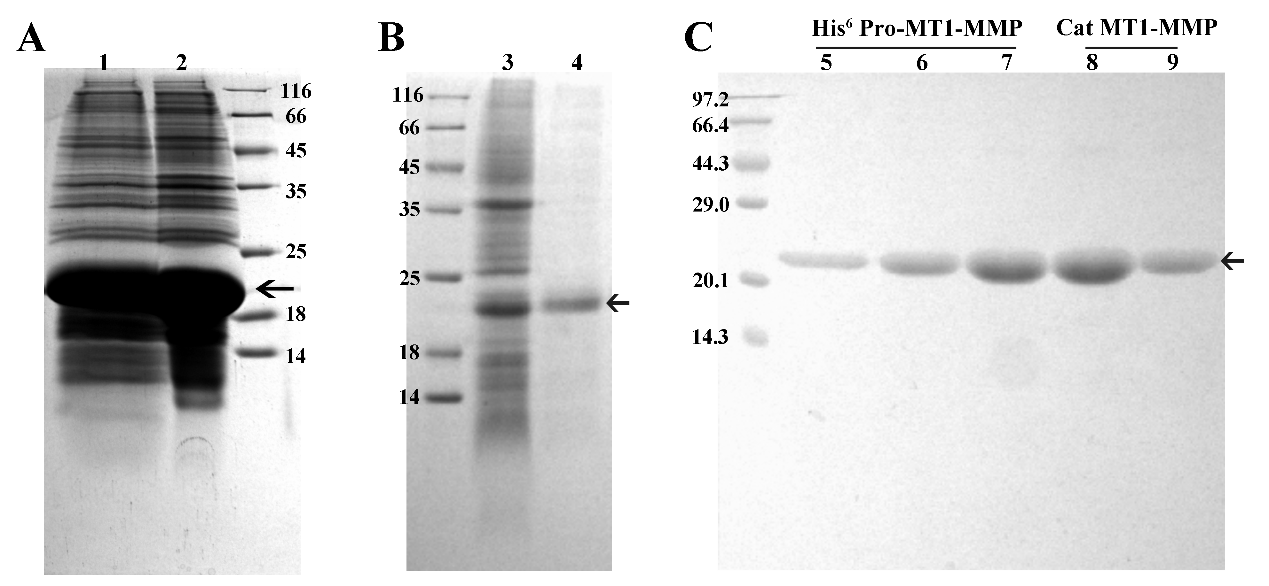


**Figure S4**. The assessment of the expression, purification and renaturation of MT1-MMP by SDS-PAGE. Arrows indicate observed positions of interested protein bands. Molecular weight (kDa) marker were labeled in numerals. Fig S4A shows the inclusion bodies fraction from the induced cultures with IPTG for 3 hours (1) and 4hours (2). Fig S4B shows the inclusion bodies before (3) and after (4) being purified by the His-tag affinity chromatography. Fig S4C displayed both pro and active form of the MT1-MMP. Lane (5-7) shows the inclusion bodies were purified by 100mM imidazole elution. Lane (8 and 9) shows the MT1-MMP with catalytic activity after refolding process. Samples in one gel derived from the same experiment and gels were processed in parallel.


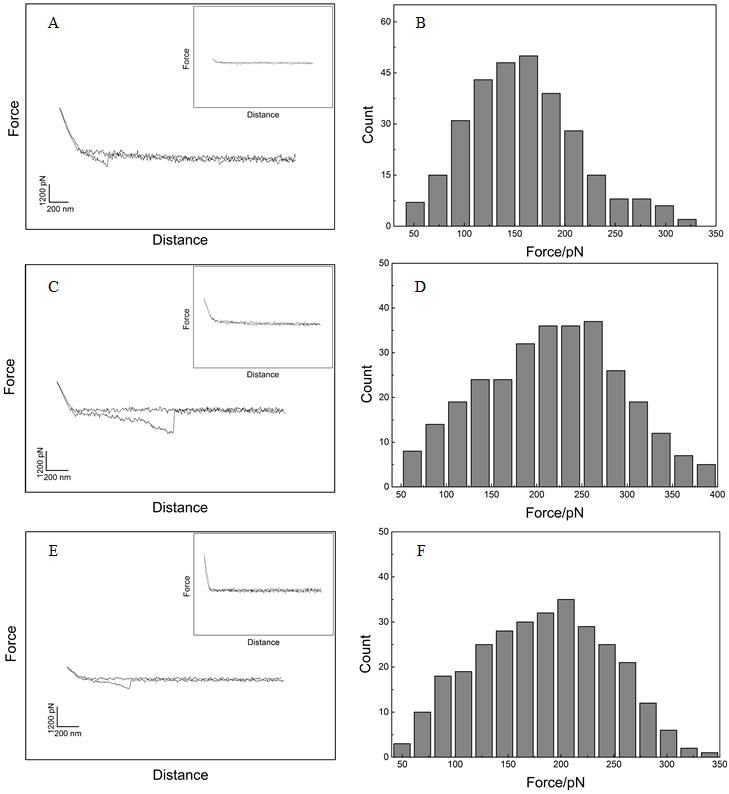


**Figure S5**. The intermolecular force measurement of optimized peptides and MT1-160p. These typical force curves showing the specific interactions between MT1-AF7p-H4R and MT1-160p(A), MT1-AF7p-H4K and MT1-160p (B) or MT1-AF7p and MT1-160p (C) at 25℃, the control experiments were done at the same condition and the force curves were shown inside each figure. The average force values were expressed as a probability in the form of histograms (figs B, D and F).


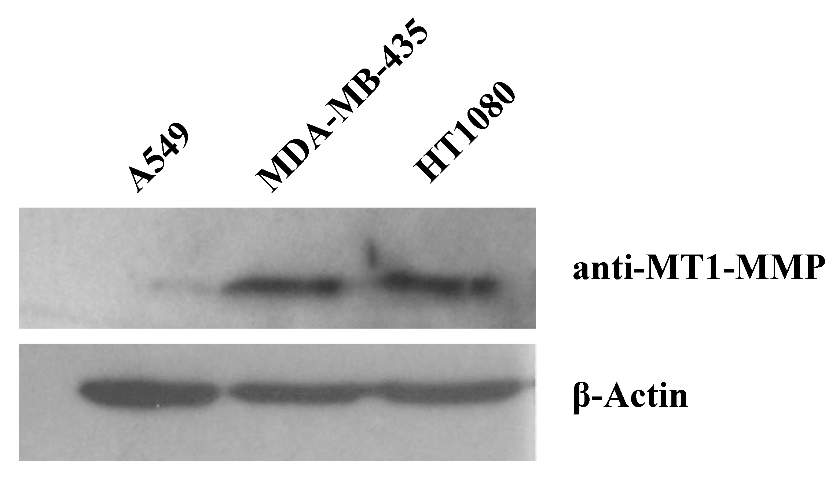


**Figure S6.** Western blot analysis of MT1-MMP expression in MDA-MB-435, HT1080 and A549 tumor cell lines. Protein samples (40 μg) from tumor cell lysate were subjected to Western blotting. Overexpressed MT1-MMP were detected in MDA-MB-435 and HT1080, compared to the low expression of MT1-MMP in A549.


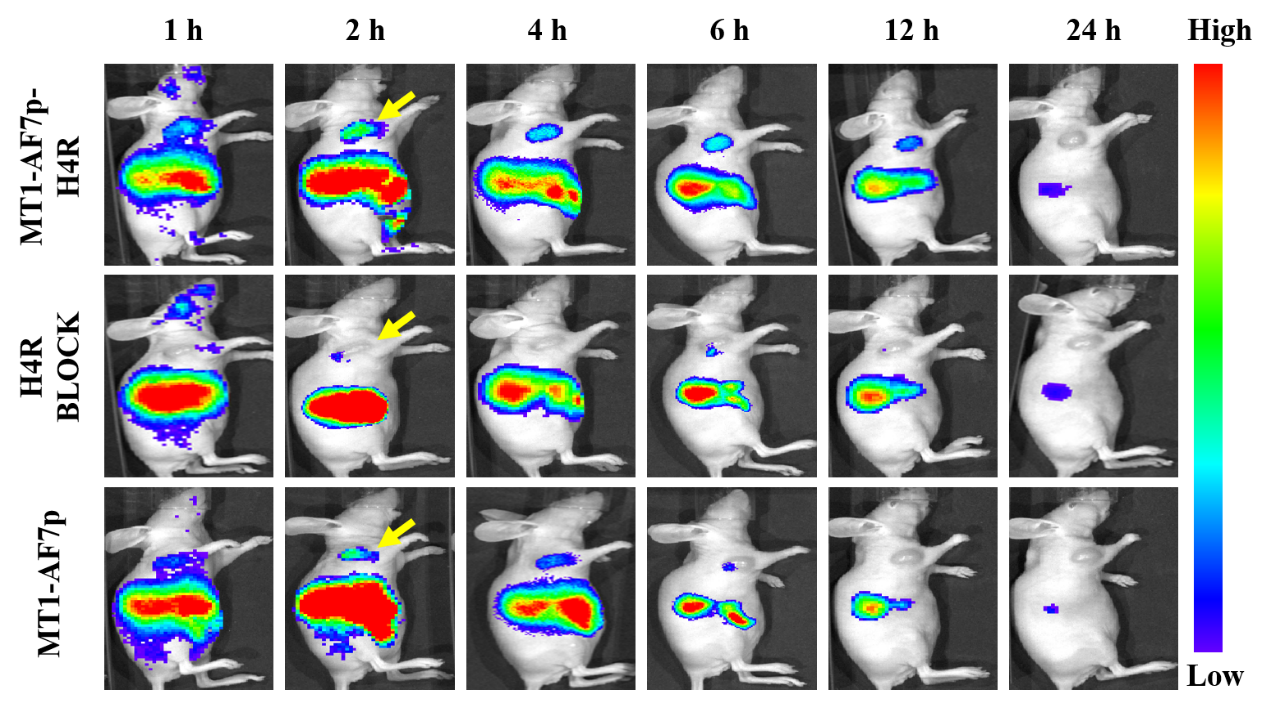


**Figure S7**. MT1-MMP sensitive optimized peptide probe exhibits enhanced signals in vivo. In vivo Near-infrared optical fluorescence imaging of MDA-MB-435 breast tumor bearing nude mice are shown after intravenous injection with Cy5.5-MT1-AF7p-H4R or Cy5.5-MT1-AF7p at different time points. Yellow arrows indicate the tumors.

**Table S1.** Energy parameter of MT1-MMP and polypeptide

| Polypeptide | RDOCK Score (kcal/mol) |
| --- | --- |
| MT1-AF7p | -62.87 |
| MT1-AF7p-H4K | -65.11 |
| MT1-AF7p-H4R | -68.99 |

**Table S2.** Hydrogen bonds parameters of MT1-AF7p with MT1-MMP

| Donors | Atom | Receptor | Atom | Distances(Å) |
| --- | --- | --- | --- | --- |
| MT1-MMP: Glu183 | HN | MT1-AF7p: Thr8 | OG1 | 2.33 |
| MT1-AF7p: His6 | HE2 | MT1-MMP: Asp212 | OD2 | 2.03 |
| MT1-AF7p: Asn7 | HD22 | MT1-MMP: Phe181 | O | 1.37 |
| MT1-AF7p: Lys9 | HZ1 | MT1-MMP: Glu183 | OE2 | 2.49 |
| MT1-AF7p: Lys9 | HZ2 | MT1-MMP: Glu183 | OE2 | 1.95 |
| MT1-AF7p: Leu12 | H1 | MT1-MMP: Tyr166 | O | 1.84 |

**Table S3.** Hydrogen bonds parameters of MT1-AF7p-H4K with MT1-MMP

| Donors | Atom | Receptor | Atom | Distances(Å) |
| --- | --- | --- | --- | --- |
| MT1-MMP: Glu183 | HN | MT1-AF7p-H4K:Thr8 | OG1 | 2.16 |
| MT1-AF7p-H4K:Lys4 | HZ1 | MT1-MMP: Gly187 | O | 2.14 |
| MT1-AF7p-H4K:Asn7 | HD22 | MT1-MMP: Phe181 | O | 1.44 |
| MT1-AF7p-H4K:Lys9 | HZ1 | MT1-MMP: Glu183 | OE2 | 2.27 |
| MT1-AF7p-H4K:Lys9 | HZ2 | MT1-MMP: Glu183 | OE2 | 1.76 |
| MT1-AF7p-H4K:Leu12 | H1 | MT1-MMP: Tyr166 | O | 2.18 |

**Table S4.** Hydrogen bonds parameters of MT1-AF7p-H4R with MT1-MMP

| Donors | Atom | Receptor | Atom | Distances(Å) |
| --- | --- | --- | --- | --- |
| MT1-MMP: Glu183 | HN | MT1-AF7p-H4R:Thr8 | OG1 | 2.36 |
| MT1-AF7p-H4R:Arg4 | HH21 | MT1-MMP: Gly187 | O | 1.42 |
| MT1-AF7p-H4R:His6 | HE2 | MT1-MMP: Asp212 | OD2 | 2.08 |
| MT1-AF7p-H4R:Asn7 | HD22 | MT1-MMP: Phe181 | O | 1.36 |
| MT1-AF7p-H4R:Lys9 | HZ1 | MT1-MMP: Glu183 | OE2 | 2.54 |
| MT1-AF7p-H4R:Lys9 | HZ2 | MT1-MMP: Glu183 | OE2 | 2.08 |
| MT1-AF7p-H4R:Leu12 | H1 | MT1-MMP: Tyr166 | O | 1.87 |

**Table S5.** Pi–pi interaction between MT1-AF7p-H4K and MT1-MMP

| End1 | End2 | Distances(Å) |
| --- | --- | --- |
| MT1-AF7p-H4K:His6 | MT1-MMP: Lys173 | 6.68 |

**Table S6.** Characteristics of Cy5.5-MT1-AF7p-H4R and Cy5.5-MT1-AF7p peptide probes

|  | Number of potential Hydrogen bonds with MT1-MMP | Size | Binding Force | Binding Constant |  | T/M ratio at 4h post-injection |
| --- | --- | --- | --- | --- | --- | --- |
| Cy5.5-MT1-AF7p-H4R | 6 pairs | 12 amino acid | 260pN | 1.75×10^5^ M^-1^ |  | 4.26 |
| Cy5.5-MT1-AF7p | 5 pairs | 12 amino acid | 150pN | 1.07×10^5^ M^-1^ |  | 1.98 |
